# Supplementary material for: The Relevance of Non‐Axiality and Low‐Lying Excited States for Slow Magnetic Relaxation in Pentagonal‐Bipyramidal Erbium(III) Complexes Probed by High‐frequency EPR
Source: Chemistry. 2025 May 15;31(33):e202500369. doi: 10.1002/chem.202500369 (PMC12161000; doi:10.1002/chem.202500369)
Supplement: Supplementary file 1 — Supporting Information [file CHEM-31-e202500369-s001.pdf]

Supporting Information: The Relevance of Non-axiality and Low-lying Excited States for Slow Magnetic Relaxation in Pentagonal-bipyramidal Erbium(III) Complexes Probed by High-frequency EPR

Jan Arneth,*[a] Lena Spillecke,[a] Changyun Koo,[a],[c] Tamara A. Bazhenova,[b] Eduard B. Yagubskii,[b] and Rüdiger Klingeler*[a]

Corresponding authors: jan.arneth@kip.uni-heidelberg.de, ruediger.klingeler@kip.uni-heidelberg.de

Table of Contents

1. Additional HF-EPR Data on Complex **(1)** 2
2. Additional HF-EPR Data on Complex **(2)** 3

1. Additional HF-EPR Data on Complex (1)

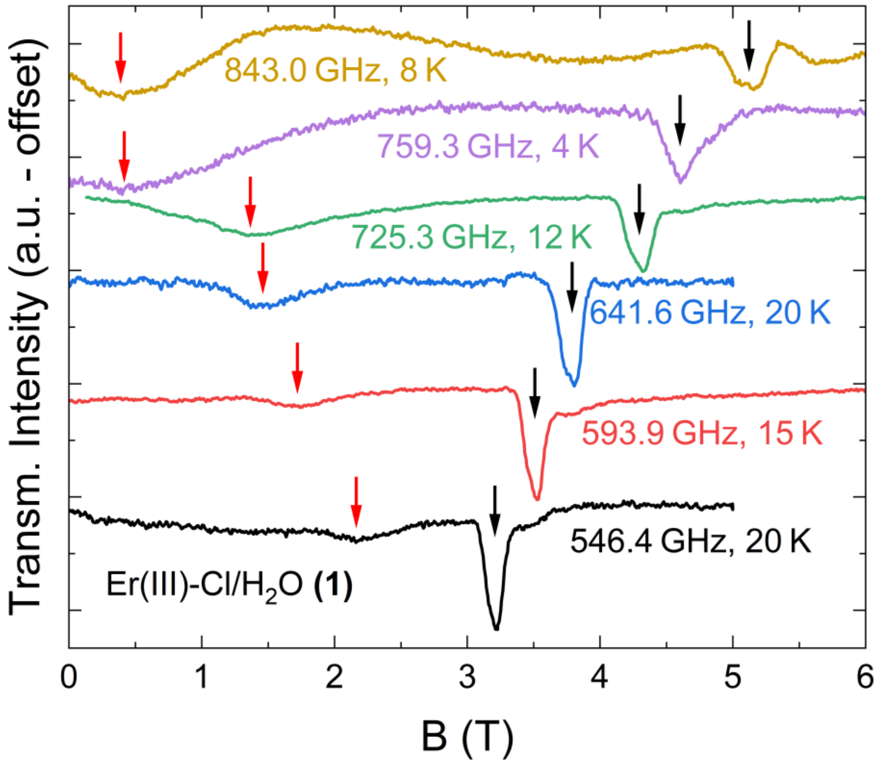

Figure S1. Loose powder HF-EPR spectra of complex **(1)** at selected fixed frequencies for *T* > 2 K. Arrows mark the read off resonance positions for the transitions arising from the ground state (black) and from excited states (red) as described in the main text.

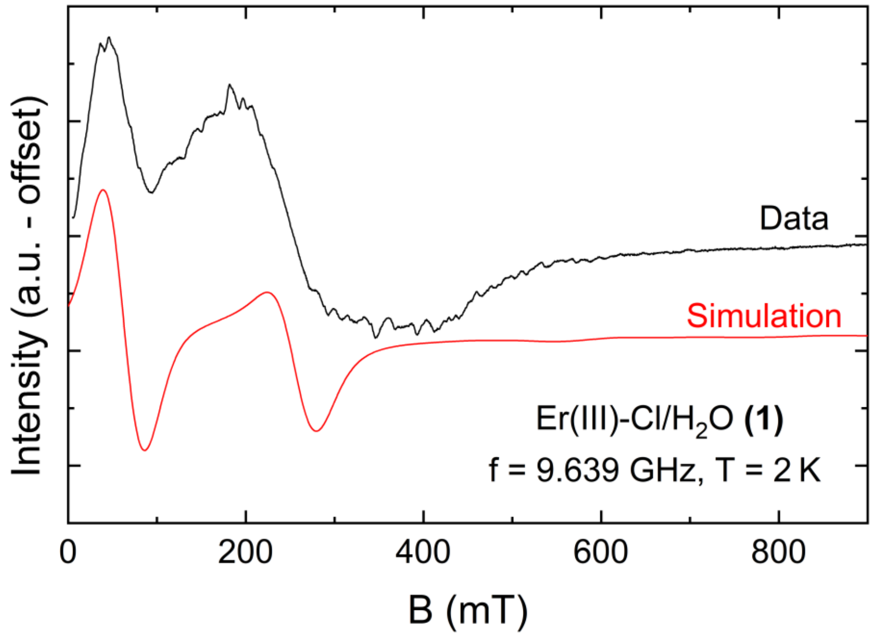

Figure S2. Fixed powder X-band spectrum of complex **(1)** at *f* = 9.639 GHz and *T* = 2 K and the corresponding simulation using a *S* = 1/2 pseudospin approximation with the parameters as described in the main text.

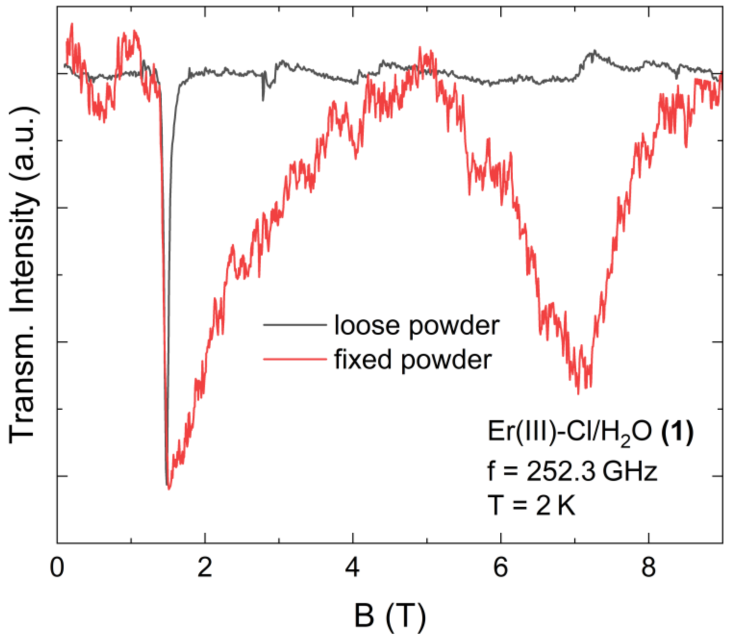

Figure S3. Comparison of loose and fixed powder HF-EPR spectra obtained at *f* = 252.3 GHz and *T* = 2 K for complex **(1)**. The fixed powder data has been scaled to match the maximum absorption with that of the loose powder data.

2. Additional HF-EPR Data on Complex (2)

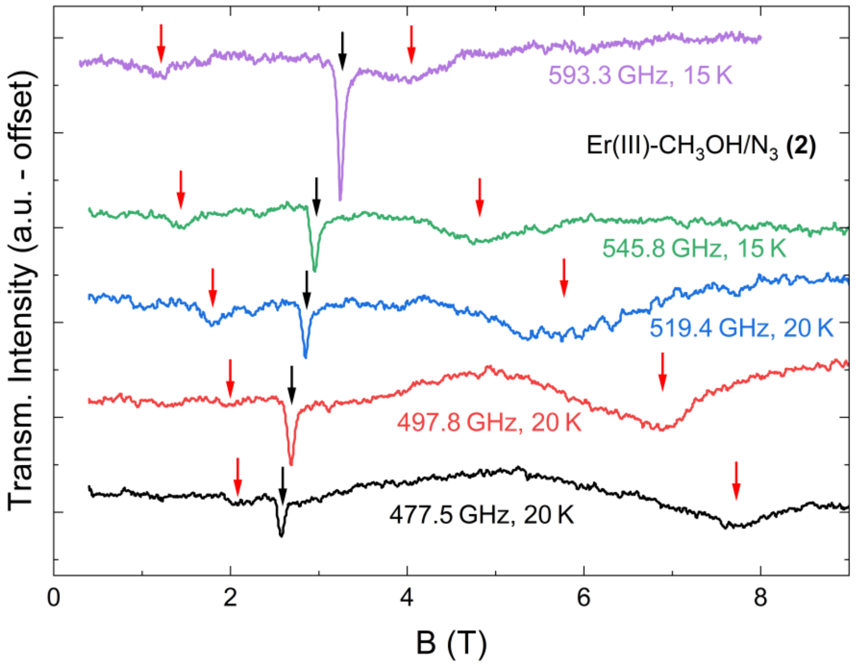

Figure S4. Loose powder HF-EPR spectra of complex **(2)** at selected fixed frequencies for *T* > 2 K. Arrows mark the read off resonance positions for the transitions arising from the ground state (black) and from excited states (red) as described in the main text.

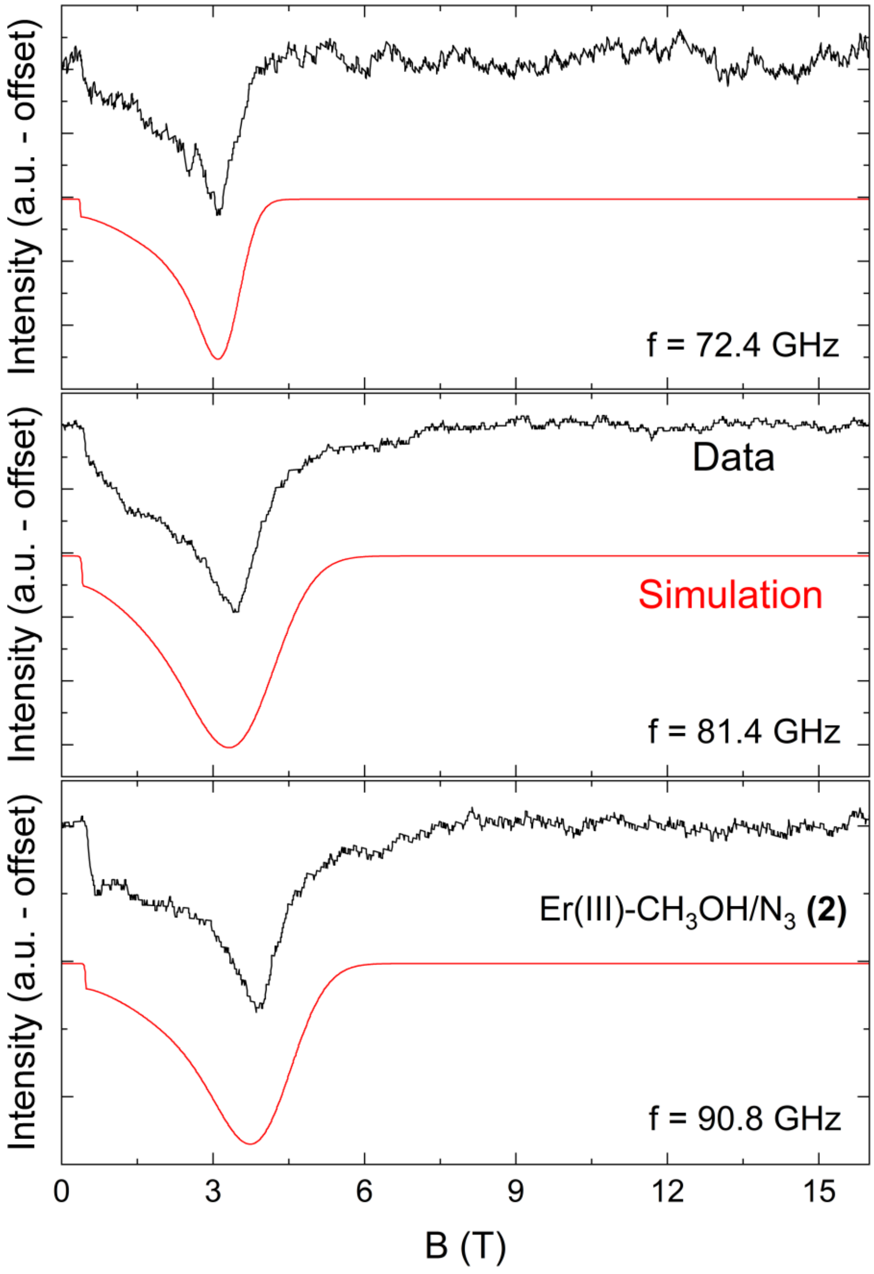

Figure S5. Fixed powder HF-EPR spectra of complex **(2)** at selected frequencies and *T* = 2K and simulations using a *S* = 1/2 pseudospin approximation with the parameters as described in the main text.

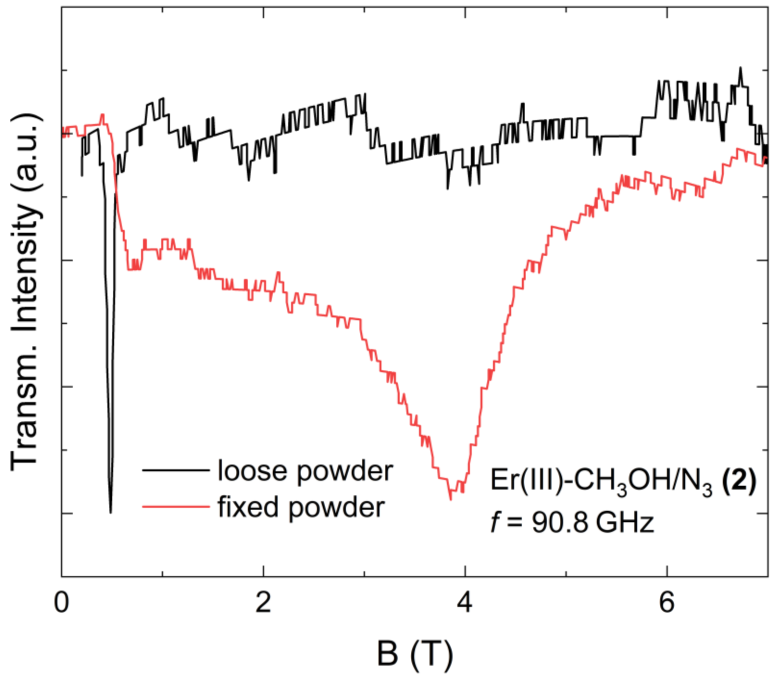

Figure S6. Comparison of loose and fixed powder HF-EPR spectra obtained at f = 90.8 GHz and T = 2 K for complex **(2)**. The fixed powder data has been scaled to match the maximum absorption with that of the loose powder data.
